# Gene-based multiple regression association testing for combined examination of common and low frequency variants in quantitative trait analysis

**DOI:** 10.3389/fgene.2013.00233

**Published:** 2013-11-12

**Authors:** Yun Joo Yoo, Lei Sun, Shelley B. Bull

**Affiliations:** ^1^Department of Mathematics Education, Seoul National UniversitySeoul, South Korea; ^2^Interdisciplinary Program in Bioinformatics, Seoul National UniversitySeoul, South Korea; ^3^Division of Biostatistics, Dalla Lana School of Public Health, University of TorontoToronto, ON, Canada; ^4^Department of Statistical Science, University of TorontoToronto, ON, Canada; ^5^Prosserman Centre for Health Research, Lunenfeld-Tanenbaum Research Institute of Mount Sinai HospitalToronto, ON, Canada

**Keywords:** genetic association analysis, multi-marker association analysis, rare variant analysis, common variant analysis, multi-bin multi-marker tests, generalized Wald test, minimum *p*-value test, indirect association

## Abstract

Multi-marker methods for genetic association analysis can be performed for common and low frequency SNPs to improve power. Regression models are an intuitive way to formulate multi-marker tests. In previous studies we evaluated regression-based multi-marker tests for common SNPs, and through identification of bins consisting of correlated SNPs, developed a multi-bin linear combination (MLC) test that is a compromise between a 1 *df* linear combination test and a multi-*df* global test. Bins of SNPs in high linkage disequilibrium (LD) are identified, and a linear combination of individual SNP statistics is constructed within each bin. Then association with the phenotype is represented by an overall statistic with *df* as many or few as the number of bins. In this report we evaluate multi-marker tests for SNPs that occur at low frequencies. There are many linear and quadratic multi-marker tests that are suitable for common or low frequency variant analysis. We compared the performance of the MLC tests with various linear and quadratic statistics in joint or marginal regressions. For these comparisons, we performed a simulation study of genotypes and quantitative traits for 85 genes with many low frequency SNPs based on HapMap Phase III. We compared the tests using (1) set of all SNPs in a gene, (2) set of common SNPs in a gene (MAF ≥ 5%), (3) set of low frequency SNPs (1% ≤ MAF < 5%). For different trait models based on low frequency causal SNPs, we found that combined analysis using all SNPs including common and low frequency SNPs is a good and robust choice whereas using common SNPs alone or low frequency SNP alone can lose power. MLC tests performed well in combined analysis except where two low frequency causal SNPs with opposing effects are positively correlated. Overall, across different sets of analysis, the joint regression Wald test showed consistently good performance whereas other statistics including the ones based on marginal regression had lower power for some situations.

## Introduction

Recently, many multi-marker methods have been developed for the analysis of rare SNPs. Among them, one class of tests is called “collapsing method” or “linear statistics” (Derkach et al., 2013). These statistics combine the individual SNP-based scores linearly with various weights. The cohort allelic sum test (CAST) (Morgenthaler and Thilly, 2007), CMC test (Li and Leal, 2008), the weighted sum test (Madsen and Browning, 2009) are well-known linear statistics. Linear statistics work well when the combined alleles are mostly deleterious or mostly protective, but when the rare variants include a substantial portion of protective and deleterious effects, they will lose power. The multi-marker tests based on a sum of squared terms are called “quadratic statistics” (Derkach et al., 2013). C-alpha test (Neale et al., 2011), SKAT (Wu et al., 2011) and SSU tests (Pan, 2009) are popular ones in this class, and are usually robust to the occurrence of deleterious and protective variants among multiple associated SNPs. Derkach et al. (2013) evaluated various linear and quadratic statistics and found that linear statistics can be powerful for specific situations but quadratic statistics have robustness to a wide range of trait model scenarios. Both Ladouceur et al. (2012) and Derkach et al. (2013) concluded that there is no single method that is consistently more powerful than other methods.

The multi-marker methods mentioned above are constructed from the marginal association analysis of the trait phenotype with each individual SNP. Alternatively, global statistics can be constructed from joint analysis of multiple SNPs in a multiple regression model. In previous studies, we developed a regression-based multi-marker method that combines linear and quadratic components using bins defined by the linkage disequilibrium (LD) patterns within a gene (Yoo et al., 2013). Regression analysis with multiple SNPs is performed and a global test statistic is constructed from the beta coefficients and associated covariance matrix. The multi-bin linear combination (MLC) statistic takes a weighted linear combination of SNPs effects within a bin of highly correlated SNPs and a quadratic function across bins as a sum of squared within-bin linear combinations. The MLC typically requires an algorithm to adjust the coding of risk and base alleles such that SNPs within a bin are positively correlated, as far as this is possible. In comparison to alternative methods, we found the MLC tests to have relatively good power and robustness under various one and two causal SNP trait models across a wide range of gene structures. Several other multi-marker statistics based on marginal regression analysis such as MinP and SSB (Pan, 2009) also compared in Yoo et al. (2013) showed good power, except for the genes with weakly correlated SNPs (that is, with low LD).

Since MLC is constructed from multi-SNP regression analysis of categorical explanatory variables, we anticipated that the MLC test would be mainly suitable for detecting association with common SNPs, assuming all SNPs, both causal and tagging, are common (MAF ≥ 5%). However, if a large sample size is available, it may be feasible to analyse low frequency variants that have 1% < MAF < 5% with the aim of detecting genes that harbor low frequency causal variants as well as common causal variants. Some multi-marker tests for rare-variant analysis, such as SSB and SSBw (Pan, 2009), can be applied for combined analysis of low and common frequency variants. There are also modified versions of rare variant tests for combined analysis such as SKAT-C (Ionita-Laza et al., 2013), and methods by Chen et al. (2012) and Curtis (2012).

In this study, we compare several gene-based multiple regression association tests including MLC tests under various trait models with low frequency causal variants. We compare different analytic strategies for study of both common and low frequency variants by formulating regression models that analyse common and low frequency SNPs together, common SNPs alone, or rare SNPs alone. We also investigate the conditions in which specific statistics tend to perform better than others.

## Materials and methods

### Regression-based framework

When there are multiple SNPs in a gene, multi-SNP analysis can be performed by multiple regression with multi-parameter hypotheses, or alternatively, by combining the results of single-SNP marginal regression analysis. Both approaches require coded genotype data. Here we assume an additive genotype model with the minor allele chosen as risk allele such that the genotype is the count of the minor allele. Suppose that *K* SNPs in a gene, denoted as *X* = (*X*_1_, *X*_2_,…, *X_K_*), have been genotyped and coded as 0, 1, or 2.

The multi-SNP joint regression model of *K* SNPs is formulated as:

$$E[Y]=\beta_{0}+\beta_{1}X_{1}+\beta_{2}X_{2}+\cdots +\beta_{K}X_{K}$$

where *E*[*Y*] is the expected value of quantitative trait *Y*. Global tests of association based on the regression analysis results are constructed using beta estimates $\hat{\beta }$ = ($\hat{\beta }$_1_, …, $\hat{\beta }$_K_) and covariance estimates Σ_*B*_ from multi-SNP multiple regression. A Wald test of the global null hypothesis of no association (β_*j*_ = 0 for all *j*) against the alternative that at least one β_*j*_ ≠ 0 is defined as

$$\text{Wald}={\hat{\beta }}^{T}\Sigma_{B}^{-1}\hat{\beta }$$

with an asymptotic null distribution that follows a chi-square distribution with *K* degrees of freedom (*df*).

The maximum value of an individual SNP test statistic can become a global statistic with proper adjustment for multiple testing. This can be done in joint regression analysis with a statistic defined as

$$\text{MinP-J}=\min\;\{p\text{-value}(Z_{1},\ldots ,Z_{K})\}.$$

where $Z=(Z_{1},\ldots ,Z_{K})=\left(\frac{{\hat{\beta }}_{1}}{\sqrt{\text{Var}({\hat{\beta }}_{1})}},\ldots ,\frac{{\hat{\beta }}_{K}}{\sqrt{\text{Var}({\hat{\beta }}_{K})}}\right)$. Because a simple Bonferroni *p*-value correction is too conservative due to the correlation between beta estimates arising from the underlying LD in the SNPs, we apply a multiple testing adjustment based on assuming a multivariate normal distribution for the test statistics (James, 1991; Conneely and Boehnke, 2007).

The marginal regression models for each of *K* different single SNPs are formulated as:

$$E[Y]=\beta_{0j}^{M}+\beta_{j}^{M}X_{j},j=1,\ldots ,K.$$

Global tests of association based on these regressions are constructed with beta estimates $\hat{\beta }$ = ($\hat{\beta }$^*M*^_1_, …, $\hat{\beta }$^*M*^_*K*_) from the marginal single-SNP regressions and covariance matrix Σ*^M^_B_*. For the latter, the covariance between marginal beta estimates from individual SNP analyses can be estimated using a GEE-type method as suggested by Pan (2009). As in the multi-marker joint regression, a global Min-P statistic in marginal analyses is

$$\text{MinP-M}=\min\;\{p\text{-value}(Z_{1}^{M},\ldots ,Z_{K}^{M})\}$$

where $(Z_{1}^{M},\ldots ,Z_{K}^{M})=\left(\frac{{\hat{\beta }}_{1}^{M}}{\sqrt{\text{Var}({\hat{\beta }}_{1}^{M})}},\ldots ,\frac{{\hat{\beta }}_{K}^{M}}{\sqrt{\text{Var}({\hat{\beta }}_{K}^{M})}}\right)$ are the test statistics for each marginal analysis.

### Gene-based multi-marker test statistics

As summarized in Table 1, we compared eleven global statistics, based on joint or marginal regression that can be applied to the genotyping data of a set of common and/or low frequency variants. In addition to the Wald and MinP tests defined above, we also consider:

1. MLC-B and MLC-Z tests
  MLC-B and MLC-Z tests are two related multi-bin multi-marker regression tests, one based on the beta coefficients and the other based on the corresponding *Z* statistics (Yoo et al., 2013). MLC tests require construction of bins with high correlation between SNP genotypes within a bin, and low correlation between SNP genotypes in different bins. Suppose *L* bins have been obtained. Then the MLC-B test is constructed using $\hat{\beta }$ = ($\hat{\beta }$_1_, …, $\hat{\beta }$_*K*_) and the covariance matrix Σ_*B*_ with a weight matrix *W_s_* and takes the form:
  $$\text{MLC-B}=(W_{s}^{T}\hat{\beta }){\left(W_{s}^{T}\Sigma_{B}W_{s}\right)}^{-1}({\hat{\beta }}^{T}W_{s})$$
  where *W_s_* = (Σ^−1^_*B*_ · *J*)(*J^T^* · Σ^−1^_*B*_ · *J*)^−1^ and *J* is a *K* by *L* matrix indicating bin assignment of the SNPs, i.e., *J*_ij_ = 1 if the *i*th SNP belongs to the *j*th bin and *J_ij_* = 0 if not.
  MLC-Z is constructed similarly using the standardized test statistic $Z_{j}={\hat{\beta }}_{j}/\sqrt{\text{Var}({\hat{\beta }}_{j})}={\hat{\beta }}_{j}/\sqrt{\Sigma_{B_{jj}}^{-1}}$ and correlation matrix Σ_*Z*_:
  $$\text{MLC-Z}=(W_{o}^{T}Z){\left(W_{o}^{T}\Sigma_{Z}W_{o}\right)}^{-1}(Z^{T}W_{o})$$
  where *W_o_* = (Σ^−1^_*Z*_ · *J*)(*J^T^* · Σ^−1^_*Z*_ · *J*)^−1^ and *J* is the same as for MLC-B.
  The asymptotic null distributions of MLC-B and MLC-Z tests are chi-square with *L df*. The Wald test is a special case of the MLC-B test where *J* is the *K* by *K* identity matrix, which corresponds to each SNP constituting a singleton bin.
2. LC-B and LC-Z tests
  At the other extreme, if one bin includes all SNPs in a gene, the MLB test reduces to a linear combination (LC) test. From the definition of MLC-B and MLC-Z, LC-B, and LC-Z tests can be formulated as:
  $$\text{LC-B}=(w_{s}^{T}\hat{\beta }){\left(w_{s}^{T}\Sigma_{B}w_{s}\right)}^{-1}({\hat{\beta }}^{T}w_{s})$$
  and
  $$\begin{array}{l} \text{LC-Z}=(w_{o}^{T}Z){\left(w_{o}^{T}\Sigma_{Z}w_{o}\right)}^{-1}(Z^{T}w_{o})\\ \text{where }w_{sj}={\left(\Sigma_{B}^{-1}\cdot J\right)}_{j}(J^{T}\cdot \Sigma_{B}^{-1}\cdot J)\\ \text{and }w_{oj}={\left(\Sigma_{Z}^{-1}\cdot J\right)}_{j}(J^{T}\cdot \Sigma_{Z}^{-1}\cdot J)\text{ with }J={\left(1,1,\ldots ,1\right)}^{T}. \end{array}$$
  The asymptotic null distributions of LC-B and LC-Z tests are chi-square with 1 *df*.
3. PC-80 test
  MLC tests reduce the dimension of testing by summing effects of correlated SNPs. A related method uses principal components of the SNP genotypes as variables in a multiple regression. Here, a gene-based test is constructed from the regression analysis of a subset of principal components (Gauderman et al., 2007), with principal components selected by a criterion of genotypic variance explained. Assuming the principal components are ordered by the size of variance explained from the largest (*P*_1_) to smallest (*P_K_*), *P*_1_,…, *P_S_* is the smallest set that explains more than 80% of the variance. Then the regression using *S* principal components is modeled as:
  $$E[Y]=\beta_{0}^{*}+\beta_{1}^{*}P_{1}+\beta_{2}^{*}P_{2}+\cdots +\beta_{S}^{*}P_{S}$$
  Using the estimated beta coefficients of principal components $\hat{\beta }$^*^ = (β^*^_1_, β^*^_2_,…,β^*^_S_) and their covariance Σ^*^_B_, the PC80 test is defined as
  $$\text{PC80}={\hat{\beta }}^{*T}\Sigma_{B}^{*\;-\;1}{\hat{\beta }}^{*}$$
  with an asymptotic null distribution that follows chi-square with *S df*. When all *K* of the principal components are included in the regression, the test statistic is the same as the Wald statistic defined above for joint regression.
4. SSB and SSBw test
  Pan (2009) proposed quadratic test statistics based on the results of marginal analysis in which squared beta coefficients are summed to form a global test with (SSBw) or without (SSB) weighting by the variance of the beta estimates. The statistics are defined as:
  $$\text{SSB}={\hat{\beta }}^{MT}{\hat{\beta }}^{M}=\sum_{i\;=\;1}^{K}{\left({\hat{\beta }}_{i}^{M}\right)}^{2}$$
  and
  $$\text{SSBw}={\left({\hat{\beta }}^{M}\right)}^{T}\text{​}{\left[\text{diag​}\left(\Sigma_{B}^{M}\right)\right]}^{-1}{\hat{\beta }}^{M}=\sum_{i\;=\;1}^{K}{\left({\hat{\beta }}_{i}^{M}\right)}^{2}/\text{Var​}\left({\hat{\beta }}_{i}^{M}\right).$$
  which have null distributions that can be approximated by a mixture of independent chi-squared components with 1 *df* (Pan, 2009).
5. SKAT
  The sequence kernel association test (SKAT) proposed by Wu et al. (2011) is a quadratic score test with flexibly devised weights that upweight rare variants. The *SKAT* statistic is constructed as
  $$\text{SKAT}=Y^{\prime }X\cdot \text{Diag​}\left(w_{1},\ldots ,w_{K}\right)\cdot X^{\prime }Y$$
  where **Y** is the *n* by 1vector of phenotypes, **X** is the *n* by *K* matrix of genotypes, and the weights are set as *w*_*i*_ = {β(*p_i_*; 1, 25)}^2^, according to the density function of the beta distribution for the MAF *p_i_* of the *k*th SNP. Asymptotically, the null distribution of *SKAT* follows a mixture distribution of independent chi-squared components with 1 *df*.

**Table 1:** **Description of multi-marker statistics investigated in this study**.

**Statistic**	**Regression model**	**Test type**	**Null distribution**	**Weights**
Wald	Joint	Quadratic	χ^2^_*K*_	Variance/covariance
MLC-B^a^	Joint	Linear/Quadratic	χ^2^_*L*_	Variance/covariance
MLC-Z^a^	Joint	Linear/Quadratic	χ^2^_*L*_	Correlation
MinP-M^a^	Marginal	N/A	MVN(0,Σ^*M*^_*B*_)	N/A
PC80^b^	Joint	Quadratic	χ^2^_*S*_	Variance/Covariance
SSB^c^	Marginal	Quadratic	∑*c_i_*χ^2^_1_	Equal weights
SSBw^c^	Marginal	Quadratic	∑*c_i_*χ^2^_1_	Variance
SKAT^d^, ^e^	Marginal	Quadratic	∑*c_i_*χ^2^_1_	{β(*p_i_*; 1, 25)}^2^
LC-B^a^	Joint	Linear	χ^2^_1_	Variance/Covariance
LC-Z^a^	Joint	Linear	χ^2^_1_	Correlation
MinP-J^a^	Joint	N/A	MVN (0, Σ_*B*_)	N/A

a *Yoo et al., 2013*.

b *Gauderman et al., 2007*.

c *Pan, 2009*.

d *Wu et al., 2011*.

e *Ionita-Laza et al., 2013*.

### Combined analysis of common and low frequency variants

To investigate the performance of gene-based tests for combined analysis of common and low frequency variants, we compared three approaches. In the first, we made no distinction between the low frequency and common variants within a gene, analysed all the variants in one multiple regression or multiple single regressions, and constructed global test statistics from all variants combined. Then we repeated analyses separately for the low frequency variants (1% < MAF < 5%) and the common variants (MAF ≥ 5%) within each gene.

For the MLC statistics, the bin construction was conducted independently in each of the three analyses. Bins can be determined by any clustering algorithm of SNPs according to the LD measure *r*. We specified *r*^2^ > 0.5 as the threshold for binning and used the LDSelect algorithm (Carlson et al., 2004) which is a greedy algorithm that constructs clusters beginning with the bigger bins first. Within each bin thus constructed, we applied the coding correction method of Pan (2009) and Wang and Elston (2007). This correction algorithm proceeds sequentially, and switches coding of 0/1 for base and risk alleles if a SNP has too many negative *r*-values with other SNPs (more than half).

We also adapted the mixture statistic SKAT-C proposed by Ionita-Laza et al. (2013) for combined analysis of rare and common variants:

$$\text{SKAT-C}=\varphi {\text{SKAT}}_{\text{rare}}+(1-\varphi ){\text{SKAT}}_{\text{common}}$$

substituting SKAT_*LF*_ for SKAT_rare_. Here each of the SKAT statistics uses a separate set of variants with different weighting schemes: *w_i_* = {β(*p_i_*; 1, 25)}^2^ for the set of low frequency variants, and *w^c^_i_* = {β(*p_i_*; 0.5, 0.5)}^2^ for the set of common variants. The mixture parameter is specified as φ = *SD*(SKAT_*LF*_)/{*SD*(SKAT_*LF*_) + *SD*(SKAT_common_)} where *SD* is the standard deviation of the SKAT statistics. Asymptotically, the null distribution of SKAT-C follows a mixture distribution of independent chi-squared components with 1 *df*.

### Indirect association for omitted causal SNPs

In the simulation study which follows below, we assume the causal variants have not been typed and are not included in the joint or marginal regressions. This corresponds, for example, to a GWAS setting with genotyping of common variants supplemented by low frequency variant genotyping that is substantially less dense than sequencing. In this case, the genotyped SNPs in the analysis set are expected to indirectly capture the causal effect, depending on how well they tag the causal variants, i.e., depending on the strength of their relationship with the causal variants. However, the regression coefficients of the genotyped SNPs will be less than that of the unobserved causal variant. In the next paragraphs, we give expressions for the expected values of the beta estimates of the markers included in the multi-SNP regression analysis using an omitted variable bias estimation procedure (Greene, 2000, pp. 334–335). We evaluate these expressions empirically for selected genes from HapMap III under trait models with one or two causal variants, and use the evaluations to help interpret the results of the simulation studies we designed to compare the gene-based test statistics.

#### Trait model with one causal variant

Suppose that *C* is the genotype variable of an unobserved causal variant not included in the analysis set of *K* SNPs with genotypes *X* = (*X*_1_, *X*_2_,…, *X_K_*). We assume the true trait model (with a mean *Y* of zero and a null intercept) is

$$Y=a_{1}C+\varepsilon \text{ where }\varepsilon \sim N(0,\sigma^{2}).$$

Then *E*[$\hat{\beta }$] in the analysis model *Y* = β_0_ + β_1_ *X*_1_ + β_2_ *X*_2_ +…+ β*_K_X_K_* is

(1) $$E[\hat{\beta }]=a_{1}\text{​}\left(d_{1},d_{2},\ldots ,d_{K}\right)$$

where *E*[$\hat{\delta }$ = ($\hat{\delta }$_1_, $\hat{\delta }$_2_, …, $\hat{\delta }$_K_)] = (*d*_1_, *d*_2_,…, *d_K_*) is the vector of expected slope coefficients from the regression model

$$C=\delta_{0}+\delta_{1}X_{1}+\delta_{2}X_{2}+\cdots +\delta_{K}X_{K}.$$

This can be easily shown from the least squares estimation equation for $\hat{\beta }$^*^ = ($\hat{\beta }$_0_, $\hat{\beta }$_1_, …, $\hat{\beta }$_*K*_):

$${\hat{\beta }}^{*}={\left(X^{\prime }X\right)}^{-1}X^{\prime }Y={\left(X^{\prime }X\right)}^{-1}X^{\prime }\text{​}\left(a_{1}C+\varepsilon \right)$$

where **X** is the *n* by (*K* + 1) genotype matrix including a column for the intercept, *Y* is the phenotype vector for *n* subjects, *C* is the *n* by 1 genotype vector for the causal SNP, and ε is the residual error vector. Equation (1) follows from *E*[$\hat{\delta }$^*^ ] = *E*[(**X**'**X**)^−1^ **X**'**C**] where $\hat{\delta }$^*^ = ($\hat{\delta }$_0_, $\hat{\delta }$_1_, $\hat{\delta }$_2_, …, $\hat{\delta }$_*K*_) and *E*[(**X**′**X**)^−1^ **X**′ε] = **0**.

For the marginal analysis of each SNP, the indirect association for each SNP in the analysis set is determined for a trait model with one causal variant *Y* = *a*_1_*C* + ε as

$$E[{\hat{\beta }}_{i}^{M}]=a_{1}\rho_{Ci}\frac{\sigma_{C}}{\sigma_{i}}=\delta_{1}\rho_{Ci}\sqrt{\frac{p_{C}(1-p_{C})}{p_{i}(1-p_{i})}}$$

where ρ_*Ci*_ is the correlation between the causal SNP and *i*th SNP in the analysis set, and *p_C_* and *p_i_* are minor allele frequency (MAF) values of the causal SNP and the *i*th SNP, respectively. Likewise, σ_*C*_ and σ_*i*_ are the standard deviations of the genotype variables for the causal SNP and the *i*th SNP, respectively.

#### Trait model with two causal variants

More generally, if the true trait model involves two causal SNPs such that

$$Y=a_{1}C_{1}+a_{2}C_{2}+\varepsilon ,$$

the vector of expected beta coefficients for *X* = (*X*_1_, *X*_2_, …, *X_K_*) is

(2) $$E[\hat{\beta }]=a_{1}(e_{1},e_{2},\ldots ,e_{K})+a_{2}(f_{1},f_{2},\ldots ,f_{K})$$

where *E*[$\hat{\lambda }$ = ($\hat{\lambda }$_1_, $\hat{\lambda }$_2_, …, $\hat{\lambda }$_*K*_)] = (*e*_1_, *e*_2_, …, *e_K_*) and *E*[$\hat{\eta }$ = ($\hat{\eta }$_1_, $\hat{\eta }$_2_, …, $\hat{\eta }$_*K*_)] = (*f*_1_, *f*_2_, …, *f_K_*) are the expected slope coefficients in the regression models for each of two causal SNP genotypes

$$\begin{array}{l} C_{1}=\lambda_{0}+\lambda_{1}X_{1}+\lambda_{2}X_{2}+\cdots +\lambda_{K}X_{K}\text{ and}\\ C_{2}=\eta_{0}+\eta_{1}X_{1}+\eta_{2}X_{2}+\cdots +\eta_{K}X_{K}. \end{array}$$

Equation (2) follows from *E*[$\hat{\lambda }$^*^] = *E*[(**X**′**X**)^−1^ **X**′**C**_1_] for $\hat{\lambda }$^*^ = ($\hat{\lambda }$_0_, $\hat{\lambda }$_1_, $\hat{\lambda }$_2_, …, $\hat{\lambda }$_*K*_) and *E*[$\hat{\eta }$] = *E*[(**X**'**X**)^−1^ **X**'**C**_2_ ] for $\hat{\eta }$^*^ = ($\hat{\eta }$_0_, $\hat{\eta }$_1_, $\hat{\eta }$_2_, …, $\hat{\eta }$_*K*_) with

$${\hat{\beta }}^{*}={\left(X^{\prime }X\right)}^{-1}X^{\prime }Y={\left(X^{\prime }X\right)}^{-1}X^{\prime }(a_{1}C_{1}+a_{1}C_{2}+\varepsilon )$$

where **C**_1_ and **C**_2_ are the *n* by 1 genotype vectors of the two causal SNPs.

For the same trait model, *Y* = *a*_1_*C*_1_ + *a*_2_*C*_2_ + ε, the expected marginal association is

$$\begin{array}{l} E[{\hat{\beta }}_{i}^{M}]=a_{1}\rho_{C_{1}i}\frac{\sigma_{C_{1}}}{\sigma_{i}}+a_{2}\rho_{C_{2}i}\frac{\sigma_{C_{2}}}{\sigma_{i}}\\ \;=\frac{1}{\sqrt{p_{i}(1-p_{i})}}(a_{1}\rho_{C_{1}i}\sqrt{p_{C_{1}}(1-p_{C_{1}})}\\ \;+a_{2}\rho_{C_{2}i}\sqrt{p_{C_{2}}(1-p_{C_{2}})}) \end{array}$$

where ρ_*C*_1_*i*_ and ρ_*C*_2_*i*_ are the correlations between each causal SNP and the *i*th SNP, *p*_*C*_1__ and *p*_*C*_2__ are MAF values of the causal SNPs, and σ_*C*_1__ and σ_*C*_2__ are the standard deviations of the genotype variables for the causal SNPs.

### Simulated data and empirical power evaluation

**Figure 1:**
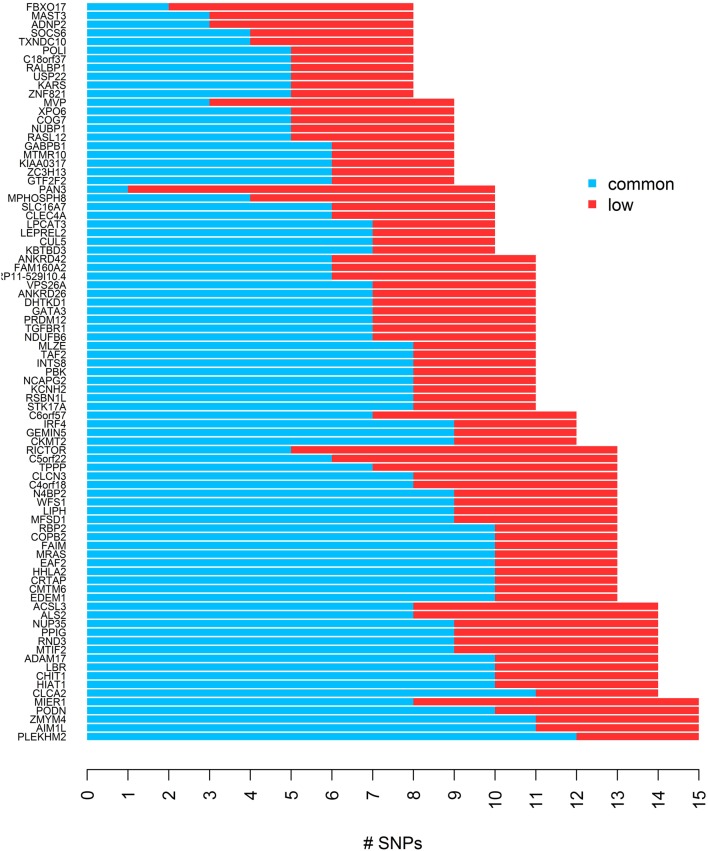
**The distribution of common and low frequency SNPs for each of 85 genes used for the simulation study**.

To evaluate the performance of different gene-based tests, we simulated quantitative trait values and genotypes in 85 gene regions which we identified in HapMap phase III, based on data for 170 individuals in the Asian population. First we excluded SNPs with MAF less than 1% from the HapMap genotype data, and then using a list of 16514 genes across 22 autosomes from the UCSC genome annotation database for NCBI hg18 Build 36.1 (http://hgdownload.soe.ucsc.edu/goldenPath/hg18/database/), we defined gene regions and constructed bins for each gene using the LDSelect algorithm with the threshold value of *r*^2^ > 0.5. We selected genes with 8–15 SNPs, and required the occurrence of 3 or more low frequency SNPs in the same bin for at least one bin, which yielded 85 genes remaining after all criteria for selection were applied. Here, we categorize SNPs with MAF ≥ 0.05 as common SNPs and SNPs with 0.01 ≤ MAF < 0.05 as low frequency SNPs. The list of genes and the distribution of low frequency and common SNPs are presented in Figure 1. The average of absolute *r* across 85 genes was 0.37 [95% CI: (0.35,0.39)] and the range was 0.17–0.59.

We considered five trait models that differed according to the number of causal SNPs, the frequency category (low frequency or common) of each causal SNP, and the direction of the causal SNP effects (Table 2). For each gene, we generated genotype data for each of *n* = 5000 people by randomly pairing haplotypes from the haplotype pool for the phased genotype data of the HapMap Asians. Then the causal SNPs were randomly assigned for each gene based on the conditions for the trait model. The low frequency causal SNPs were selected from the bin of 3 or more correlated low frequency SNPs identified at the stage of gene selection. The common causal SNPs were selected randomly from among all common SNPs. To generate quantitative trait data, we specified an additive model based on allele counts of the causal variant and a normal error model with a specific variance value. We adjusted the variance for each trait model and each gene such that the power of the Wald test is roughly 80%, to improved comparability among the genes and among the trait models. Since we limited the range of standard deviation to between 0.0001 and 100, there were several cases where the 80% power was not achieved. This procedure was repeated for each of the five trait models (that is, new genotypes were generated for each model).

**Table 2:** **Five trait models for simulation of the quantitative trait data**.

**Model label**	**Description**	Trait model parameters^*^
Model 1	One low frequency causal SNP	*a*_1_ = 1
Model 2	Two deleterious low frequency causal SNPs in the same bin	*a*_1_ = 1, *a*_2_ = 1
Model 3	Two low frequency causal SNPs, one deleterious and one protective in the same bin	*a*_1_ = 1, *a*_2_ = −1
Model 4	One common frequency causal and one low frequency causal SNP, both deleterious	*a*_1_ = 1, *a*_2_ = 1
Model 5	One deleterious common frequency causal and one protective low frequency causal SNP	*a*_1_ = 1, *a*_2_ = −1

* *The trait model is Y = a_1_ C_1_ + a_2_ C_2_ + ε where ε_~_ N(0, σ^2^). σ^2^ is specified for each gene such that the power of Wald test is about 0.8*.

We examined three analysis sets to evaluate the effects of subsetting SNPs based on MAF: (1) set of all SNPs in a gene, (2) set of common SNPs in a gene (MAF ≥ 0.05), (3) set of low frequency SNPs (0.01 ≤ MAF < 0.05). For each SNP set, joint and marginal regression analyses were performed in *N* = 1000 simulation replicates of 5000 individuals. To characterize the trait models, expected beta coefficients were summarized in various ways and averaged over genes (Table 3). In each simulation replicate, several gene-based multi-marker methods, including the MLC tests, were applied and compared. These statistics, summarized in Table 1, were chosen to include linear and quadratic statistics based on joint or marginal regression analysis. The empirical type I error and power of each statistic corresponding to a nominal 5% critical value were obtained as the proportion of data sets in which the asymptotic *p*-value was less than 0.05 among 1000 replicates.

**Table 3:** **Summary of expected beta coefficients for joint and marginal regression analysis using three analysis sets averaged over 85 genes**.

**Model**	**Analysis set**	**Method**	**Percentage of |β| > 0.5^a^**	**Sum of^b^ β**	**Sum of^c^ |β|**	**Mean of^d^ β**	**Mean of^e^ |β|**
**1**	All SNPs	Joint	17.1	0.64	1.70	0.07	0.19
		Marginal	22.9	1.99	2.33	0.22	0.25
	Common SNPs	Joint	24.5	0.06	1.80	0.02	0.26
		Marginal	4.2	0.33	0.64	0.06	0.10
	Low frequency	Joint	39.7	0.92	1.15	0.35	0.41
	SNPs	Marginal	74.3	1.82	1.87	0.66	0.68
**2**	All SNPs	Joint	29.8	0.83	3.33	0.11	0.40
		Marginal	23.3	2.63	3.27	0.31	0.38
	Common SNPs	Joint	35.5	0.18	3.34	0.05	0.50
		Marginal	11.0	0.65	1.25	0.12	0.20
	Low frequency	Joint	73.9	1.65	1.77	1.07	1.12
	SNPs	Marginal	79.3	2.15	2.20	1.23	1.24
**3**	All SNPs	Joint	13.6	−0.03	1.58	0.002	0.18
		Marginal	2.9	−0.07	0.45	−0.01	0.05
	Common SNPs	Joint	9.8	−0.01	0.99	0.003	0.14
		Marginal	0.2	0.003	0.19	0.002	0.03
	Low frequency	Joint	13.0	−0.05	0.38	−0.03	0.15
	SNPs	Marginal	9.0	−0.06	0.28	−0.03	0.13
**4**	All SNPs	Joint	32.8	1.23	3.45	0.15	0.40
		Marginal	61.2	4.30	5.87	0.51	0.68
	Common SNPs	Joint	38.8	0.54	2.73	0.12	0.46
		Marginal	56.2	2.12	3.48	0.37	0.57
	Low frequency	Joint	51.9	1.24	1.84	0.44	0.63
	SNPs	Marginal	72.8	2.36	2.58	0.82	0.89
**5**	All SNPs	Joint	33.7	0.22	3.46	0.02	0.41
		Marginal	53.0	−0.05	4.78	−0.02	0.55
	Common SNPs	Joint	39.3	−0.26	2.82	−0.05	0.47
		Marginal	49.8	−1.30	3.09	−0.22	0.50
	Low frequency	Joint	51.3	0.67	1.57	0.27	0.54
	SNPs	Marginal	60.2	1.27	1.96	0.50	0.69

a *Average of percentages of absolute beta coefficients that are greater than 0.5 within each gene*.

b *Average of sum of all beta coefficients within each gene*.

c *Average of sum of all absolute beta coefficients within each gene*.

d *Average of mean of all beta coefficients within each gene*.

e *Average of mean of all absolute beta coefficients within each gene*.

## Results

### Comparisons among analysis of three SNP sets

**Figure 2:**
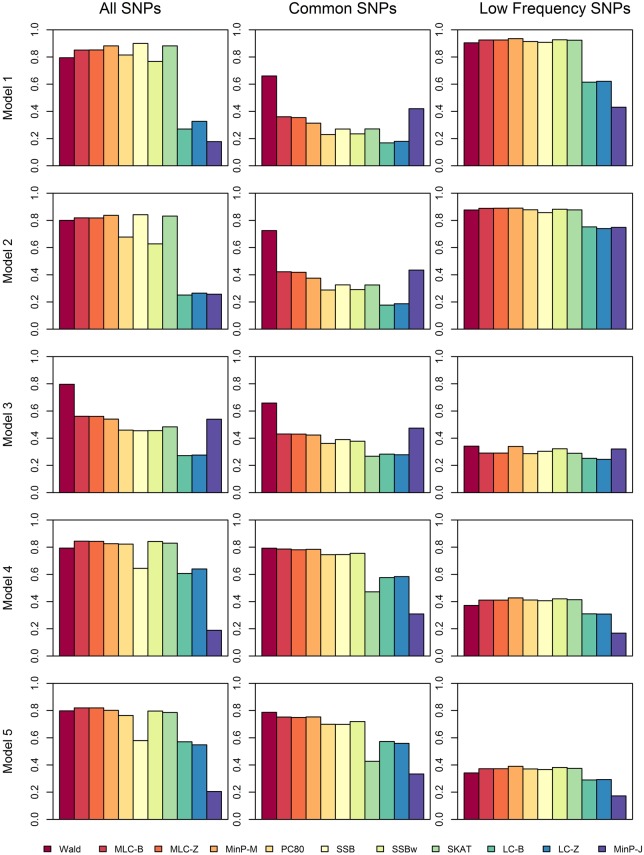
**Averaged empirical power of gene-based tests for three analysis sets obtained under five different trait models**.

**Figure 3:**
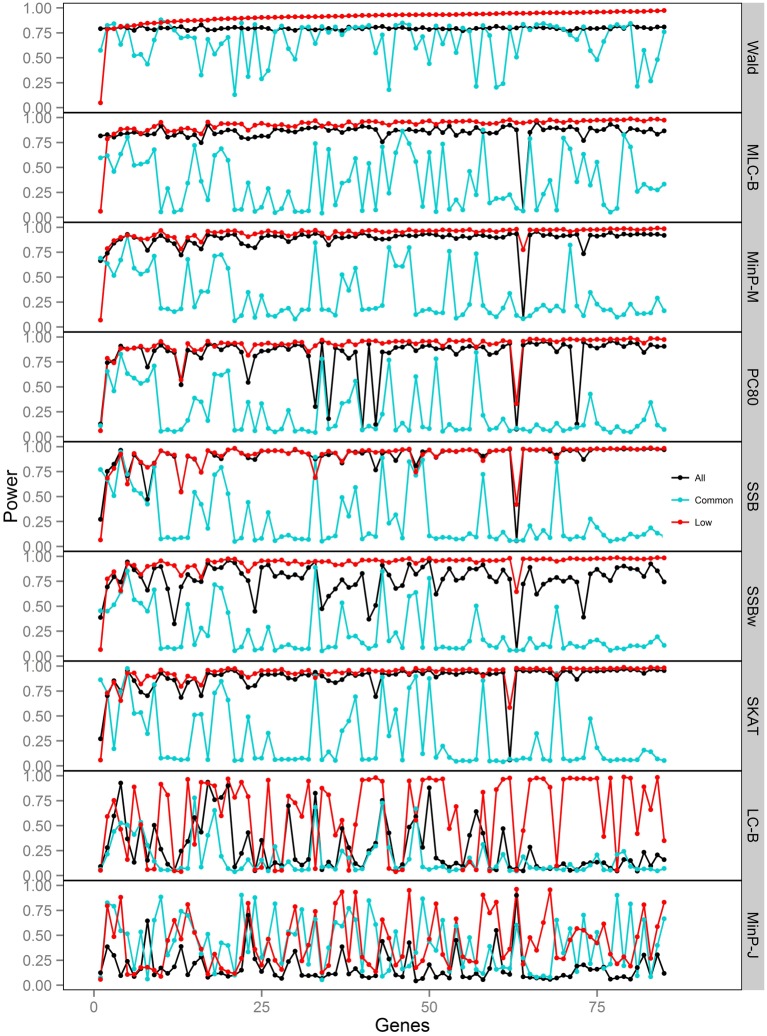
**Power of gene-based tests using three analysis sets of SNPs for 85 genes under trait *Model 1***. Genes are ordered along the horizontal axis according to the empirical power of Wald test using only low frequency SNPs.

**Figure 4:**
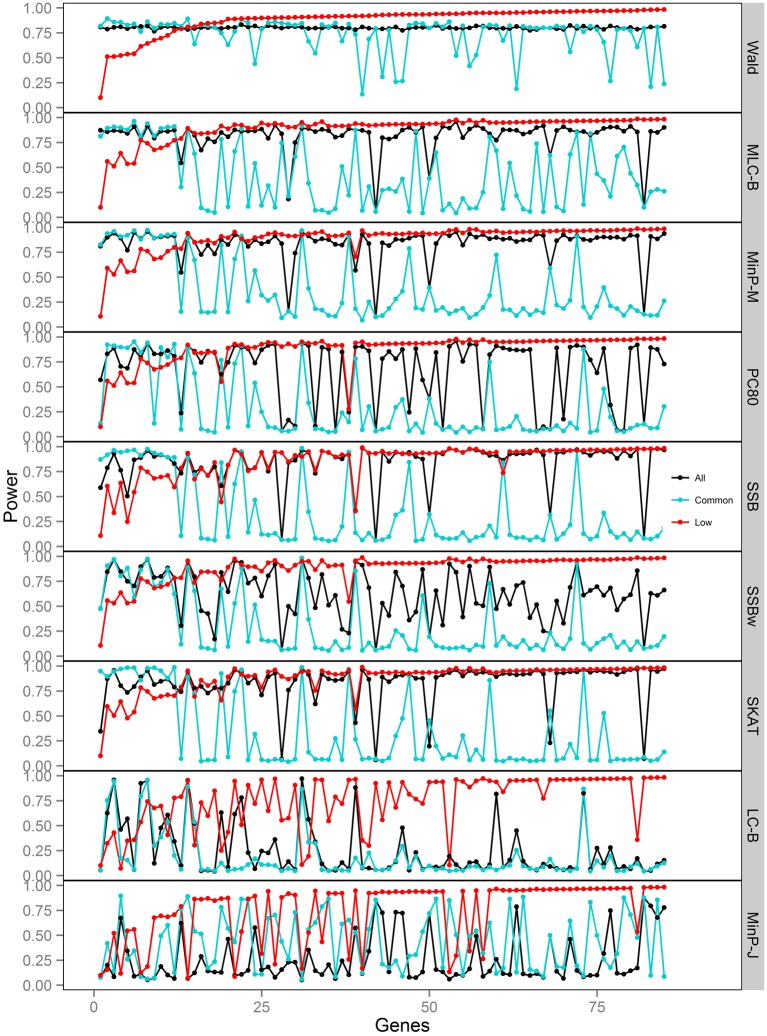
**Power of gene-based tests using three analysis sets of SNPs for 85 genes under trait *Model 2.*** Genes are ordered along the horizontal axis according to the empirical power of Wald test using only low frequency SNPs.

**Figure 5:**
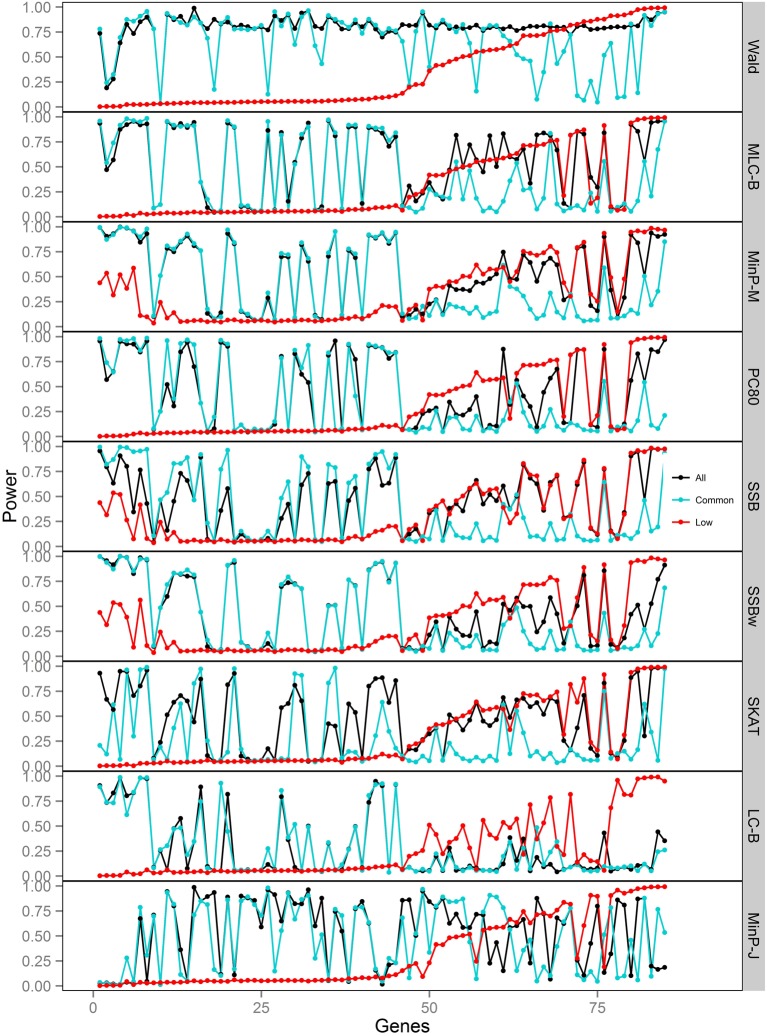
**Power of gene-based tests using three analysis sets of SNPs for 85 genes under trait *Model 3.*** Genes are ordered along the horizontal axis according to the empirical power of Wald test using only low frequency SNPs.

**Figure 6:**
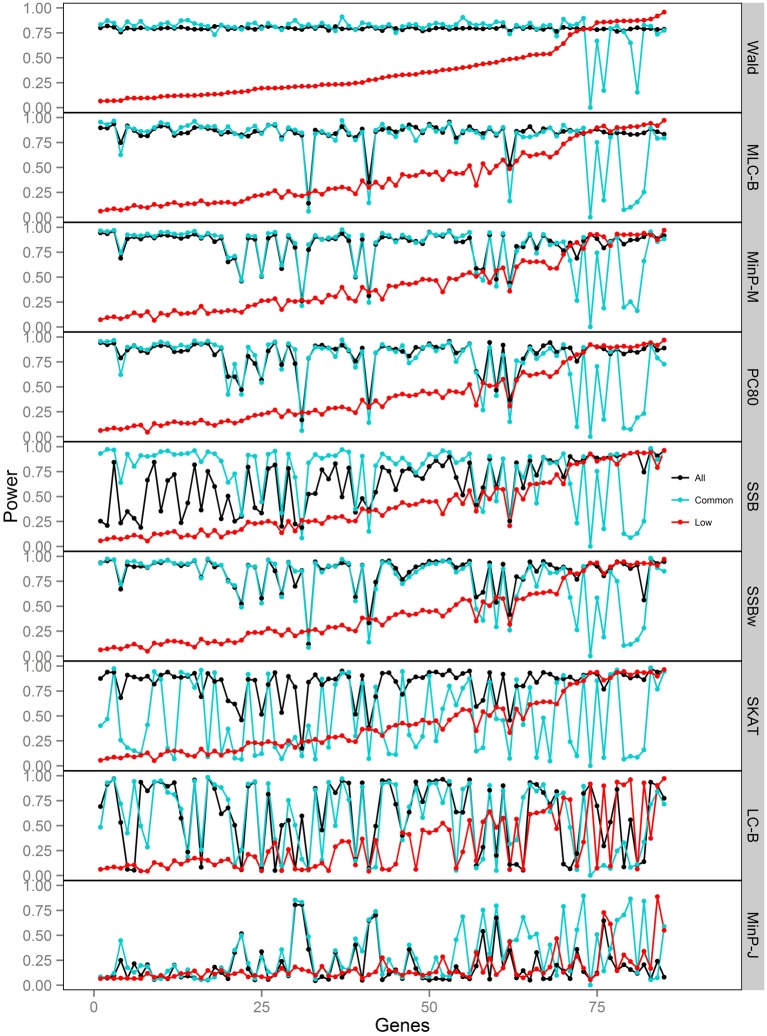
**Power of gene-based tests using three analysis sets of SNPs for 85 genes under trait *Model 4.*** Genes are ordered along the horizontal axis according to the empirical power of Wald test using only low frequency SNPs.

**Figure 7:**
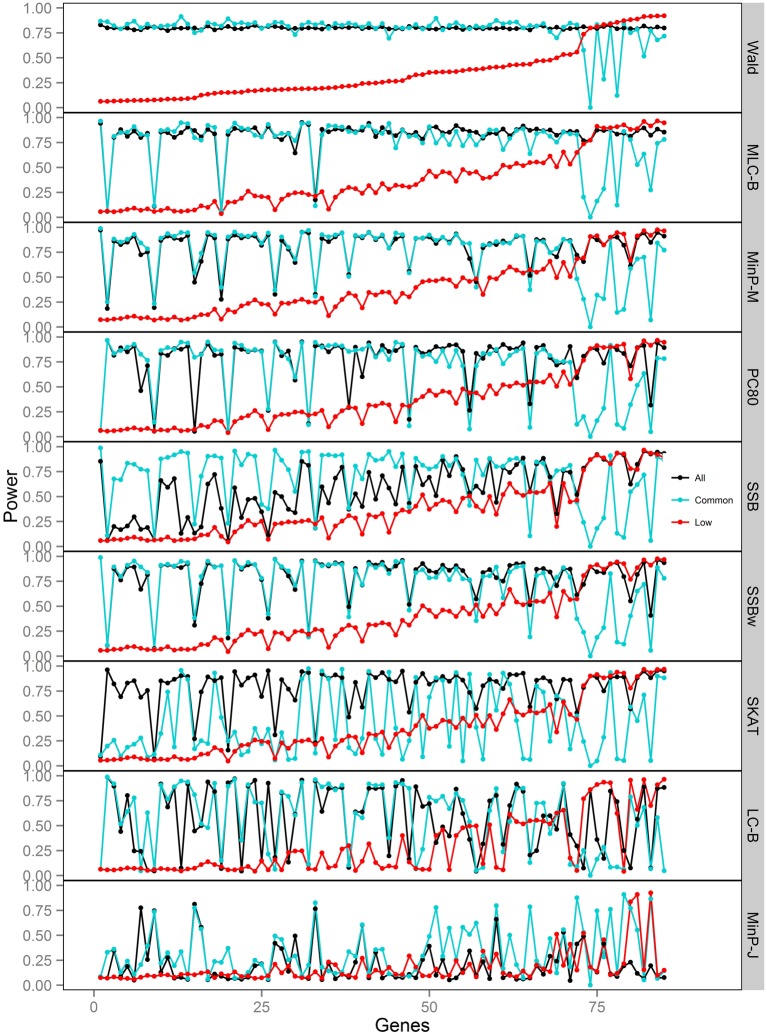
**Power of gene-based tests using three analysis sets of SNPs for 85 genes under trait *Model 5.*** Genes are ordered along the horizontal axis according to the empirical power of Wald test using only low frequency SNPs.

**Figure 8:**
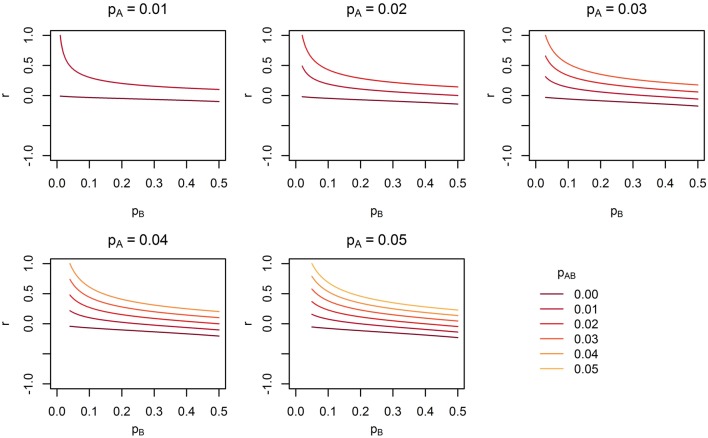
**The range of linkage disequilibrium measure *r* (correlation coefficient) with a given MAF of rare SNP *A* for range of MAF of SNP *B***. *p_A_* is the MAF of SNP *A*, *p_B_* is the MAF of SNP *B*, and *p_AB_* is the haplotype frequency consisting of rare alleles of SNP *A* and *B*.

We compared the power of gene-based tests obtained from three analysis sets for each gene panel: (1) set of all SNPs in a gene (common and low frequency), (2) common SNPs only, (3) low frequency (LF) SNPs only. We report the type I error evaluation and empirical power values averaged across the 85 genes with corresponding confidence intervals (Figure 2, Tables 4, 5). Comparison plots of the three analysis sets for most of test statistics (except MLC-Z and LC-Z since they are virtually equal to MLC-B and LC-B) also display the power values for each of the 85 genes (Figures 3–8).

**Table 4:** **Empirical type I error of gene-based statistics (*N* = 1000 replicates) at the 0.05 level for three analysis sets, averaged over 85 genes**.

		**All**		**Common**		**Low frequency**	
**Model**	**Statistics**	**Average**	**95% CI**	**Average**	**95% CI**	**Average**	**95% CI**
**1**	Wald	0.055	(0.054, 0.057)	0.054	(0.052, 0.055)	0.053	(0.052, 0.055)
	MLC-B	0.054	(0.052, 0.055)	0.053	(0.052, 0.054)	0.053	(0.051, 0.054)
	MLC-Z	0.053	(0.052, 0.055)	0.053	(0.051, 0.054)	0.053	(0.052, 0.055)
	MinP-M	0.054	(0.052, 0.057)	0.057	(0.055, 0.060)	0.063	(0.061, 0.065)
	PC80	0.053	(0.051, 0.055)	0.054	(0.052, 0.055)	0.053	(0.051, 0.054)
	SSB	0.055	(0.054, 0.057)	0.053	(0.051, 0.054)	0.055	(0.053, 0.056)
	SSBw	0.054	(0.053, 0.056)	0.053	(0.052, 0.055)	0.055	(0.053, 0.056)
	SKAT	0.054	(0.052, 0.055)	0.053	(0.051, 0.054)	0.053	(0.051, 0.054)
	LC-B	0.052	(0.050, 0.054)	0.053	(0.052, 0.055)	0.052	(0.051, 0.054)
	LC-Z	0.052	(0.051, 0.054)	0.053	(0.052, 0.054)	0.052	(0.051, 0.054)
	MinP-J	0.050	(0.049, 0.052)	0.053	(0.051, 0.055)	0.059	(0.058, 0.061)
**2**	Wald	0.049	(0.048, 0.051)	0.049	(0.047, 0.050)	0.050	(0.048, 0.051)
	MLC-B	0.049	(0.048, 0.050)	0.050	(0.048, 0.051)	0.049	(0.048, 0.051)
	MLC-Z	0.049	(0.047, 0.050)	0.050	(0.048, 0.051)	0.049	(0.048, 0.051)
	MinP-M	0.051	(0.049, 0.054)	0.052	(0.050, 0.054)	0.053	(0.052, 0.055)
	PC80	0.048	(0.047, 0.050)	0.049	(0.048, 0.051)	0.049	(0.048, 0.051)
	SSB	0.049	(0.048, 0.051)	0.048	(0.047, 0.050)	0.050	(0.049, 0.052)
	SSBw	0.049	(0.048, 0.051)	0.049	(0.048, 0.051)	0.051	(0.049, 0.052)
	SKAT	0.048	(0.047, 0.049)	0.048	(0.047, 0.050)	0.049	(0.048, 0.050)
	LC-B	0.048	(0.047, 0.050)	0.049	(0.047, 0.050)	0.049	(0.048, 0.051)
	LC-Z	0.048	(0.046, 0.049)	0.049	(0.047, 0.051)	0.050	(0.048, 0.051)
	MinP-J	0.047	(0.046, 0.049)	0.049	(0.047, 0.050)	0.052	(0.051, 0.054)
**3**	Wald	0.049	(0.047, 0.051)	0.049	(0.048, 0.051)	0.049	(0.048, 0.050)
	MLC-B	0.050	(0.049, 0.052)	0.050	(0.049, 0.052)	0.049	(0.047, 0.050)
	MLC-Z	0.050	(0.049, 0.052)	0.050	(0.049, 0.052)	0.049	(0.047, 0.050)
	MinP-M	0.055	(0.052, 0.058)	0.055	(0.053, 0.057)	0.053	(0.051, 0.055)
	PC80	0.050	(0.049, 0.052)	0.050	(0.049, 0.052)	0.049	(0.047, 0.050)
	SSB	0.051	(0.050, 0.053)	0.052	(0.050, 0.053)	0.051	(0.049, 0.052)
	SSBw	0.052	(0.050, 0.053)	0.052	(0.050, 0.053)	0.050	(0.049, 0.052)
	SKAT	0.050	(0.048, 0.051)	0.050	(0.048, 0.051)	0.048	(0.047, 0.050)
	LC-B	0.051	(0.049, 0.052)	0.051	(0.049, 0.053)	0.049	(0.048, 0.050)
	LC-Z	0.050	(0.049, 0.052)	0.051	(0.049, 0.052)	0.049	(0.047, 0.050)
	MinP-J	0.047	(0.045, 0.048)	0.047	(0.046, 0.049)	0.050	(0.048, 0.051)
**4**	Wald	0.048	(0.047, 0.050)	0.048	(0.047, 0.050)	0.049	(0.048, 0.051)
	MLC-B	0.049	(0.048, 0.050)	0.049	(0.048, 0.051)	0.049	(0.048, 0.051)
	MLC-Z	0.049	(0.048, 0.050)	0.049	(0.048, 0.051)	0.049	(0.048, 0.050)
	MinP-M	0.050	(0.048, 0.052)	0.054	(0.052, 0.056)	0.058	(0.057, 0.060)
	PC80	0.050	(0.048, 0.051)	0.049	(0.048, 0.051)	0.049	(0.048, 0.051)
	SSB	0.050	(0.049, 0.052)	0.050	(0.048, 0.051)	0.051	(0.050, 0.052)
	SSBw	0.051	(0.049, 0.052)	0.050	(0.048, 0.052)	0.051	(0.050, 0.052)
	SKAT	0.050	(0.048, 0.051)	0.049	(0.048, 0.051)	0.049	(0.048, 0.051)
	LC-B	0.050	(0.048, 0.051)	0.048	(0.047, 0.050)	0.049	(0.048, 0.051)
	LC-Z	0.050	(0.049, 0.052)	0.048	(0.047, 0.050)	0.050	(0.048, 0.051)
	MinP-J	0.046	(0.044, 0.047)	0.049	(0.047, 0.051)	0.054	(0.052, 0.056)
**5**	Wald	0.048	(0.047, 0.050)	0.049	(0.047, 0.050)	0.049	(0.047, 0.050)
	MLC-B	0.048	(0.047, 0.050)	0.049	(0.047, 0.050)	0.049	(0.047, 0.051)
	MLC-Z	0.048	(0.047, 0.050)	0.049	(0.048, 0.050)	0.049	(0.047, 0.051)
	MinP-M	0.050	(0.047, 0.052)	0.053	(0.051, 0.055)	0.059	(0.057, 0.061)
	PC80	0.048	(0.047, 0.050)	0.049	(0.048, 0.051)	0.049	(0.047, 0.051)
	SSB	0.051	(0.049, 0.052)	0.049	(0.048, 0.051)	0.051	(0.049, 0.052)
	SSBw	0.050	(0.049, 0.052)	0.050	(0.048, 0.051)	0.051	(0.049, 0.053)
	SKAT	0.049	(0.047, 0.050)	0.048	(0.047, 0.050)	0.049	(0.047, 0.051)
	LC-B	0.050	(0.048, 0.051)	0.049	(0.048, 0.051)	0.049	(0.048, 0.051)
	LC-Z	0.050	(0.048, 0.052)	0.049	(0.048, 0.051)	0.049	(0.048, 0.051)
	MinP-J	0.047	(0.046, 0.049)	0.049	(0.047, 0.051)	0.055	(0.053, 0.057)

**Table 5:** **Empirical power of gene-based statistics (*N* = 1000 replicates) at the 0.05 level for three analysis sets, averaged over 85 genes**.

		**All**		**Common**		**Low frequency**	
**Model**	**Statistic**	**Average**	**95% CI**	**Average**	**95% CI**	**Average**	**95% CI**
**1**	Wald	0.79	(0.79, 0.80)	0.66	(0.62, 0.70)	0.90	(0.88, 0.93)
	MLC-B	0.85	(0.83, 0.87)	0.36	(0.30, 0.42)	0.93	(0.90, 0.95)
	MLC-Z	0.85	(0.83, 0.87)	0.35	(0.30, 0.41)	0.93	(0.90, 0.95)
	MinP-M	0.88	(0.86, 0.90)	0.31	(0.26, 0.37)	0.93	(0.91, 0.96)
	PC80	0.81	(0.77, 0.86)	0.23	(0.18, 0.28)	0.91	(0.89, 0.94)
	SSB	0.90	(0.87, 0.93)	0.27	(0.21, 0.33)	0.91	(0.88, 0.94)
	SSBw	0.77	(0.73, 0.80)	0.23	(0.18, 0.28)	0.93	(0.90, 0.95)
	SKAT	0.88	(0.85, 0.91)	0.27	(0.21, 0.34)	0.92	(0.90, 0.95)
	LC-B	0.27	(0.22, 0.32)	0.17	(0.13, 0.21)	0.62	(0.53, 0.70)
	LC-Z	0.33	(0.27, 0.39)	0.18	(0.14, 0.22)	0.62	(0.54, 0.70)
	MinP-J	0.18	(0.14, 0.21)	0.42	(0.36, 0.48)	0.43	(0.37, 0.49)
**2**	Wald	0.80	(0.80, 0.80)	0.73	(0.68, 0.77)	0.88	(0.85, 0.91)
	MLC-B	0.82	(0.78, 0.85)	0.42	(0.35, 0.49)	0.89	(0.86, 0.92)
	MLC-Z	0.82	(0.78, 0.85)	0.42	(0.35, 0.49)	0.89	(0.86, 0.92)
	MinP-M	0.84	(0.80, 0.87)	0.38	(0.31, 0.44)	0.89	(0.86, 0.92)
	PC80	0.68	(0.61, 0.74)	0.29	(0.22, 0.36)	0.88	(0.84, 0.91)
	SSB	0.84	(0.80, 0.89)	0.33	(0.25, 0.40)	0.86	(0.82, 0.90)
	SSBw	0.63	(0.58, 0.68)	0.29	(0.22, 0.36)	0.88	(0.85, 0.91)
	SKAT	0.83	(0.79, 0.88)	0.33	(0.25, 0.41)	0.88	(0.84, 0.91)
	LC-B	0.25	(0.19, 0.31)	0.18	(0.13, 0.23)	0.75	(0.69, 0.81)
	LC-Z	0.26	(0.20, 0.33)	0.19	(0.13, 0.24)	0.74	(0.68, 0.80)
	MinP-J	0.26	(0.21, 0.31)	0.43	(0.37, 0.50)	0.75	(0.68, 0.81)
**3**	Wald	0.80	(0.77, 0.83)	0.66	(0.60, 0.72)	0.34	(0.27, 0.42)
	MLC-B	0.56	(0.49, 0.63)	0.43	(0.35, 0.51)	0.29	(0.22, 0.36)
	MLC-Z	0.56	(0.49, 0.63)	0.43	(0.35, 0.51)	0.29	(0.22, 0.36)
	MinP-M	0.54	(0.47, 0.61)	0.42	(0.35, 0.50)	0.34	(0.27, 0.40)
	PC80	0.46	(0.38, 0.54)	0.36	(0.28, 0.44)	0.29	(0.22, 0.36)
	SSB	0.45	(0.39, 0.52)	0.39	(0.31, 0.47)	0.30	(0.24, 0.37)
	SSBw	0.46	(0.38, 0.53)	0.38	(0.30, 0.45)	0.32	(0.26, 0.39)
	SKAT	0.48	(0.42, 0.55)	0.27	(0.20, 0.33)	0.29	(0.22, 0.36)
	LC-B	0.27	(0.21, 0.34)	0.28	(0.22, 0.35)	0.25	(0.19, 0.32)
	LC-Z	0.28	(0.21, 0.34)	0.28	(0.21, 0.34)	0.24	(0.18, 0.31)
	MinP-J	0.54	(0.47, 0.61)	0.47	(0.40, 0.55)	0.32	(0.25, 0.39)
**4**	Wald	0.79	(0.79, 0.80)	0.79	(0.76, 0.82)	0.37	(0.32, 0.43)
	MLC-B	0.84	(0.82, 0.87)	0.79	(0.73, 0.84)	0.41	(0.35, 0.47)
	MLC-Z	0.84	(0.82, 0.87)	0.78	(0.73, 0.83)	0.41	(0.35, 0.47)
	MinP-M	0.83	(0.79, 0.86)	0.78	(0.73, 0.84)	0.43	(0.37, 0.49)
	PC80	0.82	(0.79, 0.86)	0.74	(0.69, 0.80)	0.41	(0.35, 0.47)
	SSB	0.64	(0.59, 0.70)	0.75	(0.69, 0.80)	0.41	(0.35, 0.47)
	SSBw	0.84	(0.81, 0.88)	0.75	(0.70, 0.81)	0.42	(0.36, 0.48)
	SKAT	0.83	(0.80, 0.86)	0.47	(0.40, 0.55)	0.41	(0.35, 0.48)
	LC-B	0.61	(0.53, 0.68)	0.58	(0.50, 0.65)	0.31	(0.25, 0.37)
	LC-Z	0.64	(0.57, 0.71)	0.58	(0.51, 0.66)	0.31	(0.25, 0.37)
	MinP-J	0.19	(0.15, 0.23)	0.31	(0.25, 0.36)	0.17	(0.14, 0.20)
**5**	Wald	0.80	(0.80, 0.80)	0.79	(0.76, 0.82)	0.34	(0.29, 0.40)
	MLC-B	0.82	(0.78, 0.86)	0.75	(0.70, 0.80)	0.37	(0.31, 0.43)
	MLC-Z	0.82	(0.78, 0.86)	0.75	(0.70, 0.80)	0.37	(0.31, 0.43)
	MinP-M	0.80	(0.76, 0.84)	0.75	(0.70, 0.81)	0.39	(0.33, 0.45)
	PC80	0.76	(0.71, 0.82)	0.70	(0.64, 0.76)	0.37	(0.31, 0.43)
	SSB	0.58	(0.52, 0.63)	0.70	(0.64, 0.76)	0.37	(0.31, 0.43)
	SSBw	0.80	(0.75, 0.84)	0.72	(0.66, 0.78)	0.38	(0.32, 0.44)
	SKAT	0.79	(0.74, 0.83)	0.43	(0.36, 0.50)	0.37	(0.31, 0.43)
	LC-B	0.57	(0.50, 0.64)	0.57	(0.50, 0.64)	0.29	(0.23, 0.35)
	LC-Z	0.55	(0.48, 0.62)	0.56	(0.49, 0.63)	0.29	(0.23, 0.36)
	MinP-J	0.20	(0.16, 0.25)	0.33	(0.28, 0.39)	0.17	(0.14, 0.21)

Empirical type I error, averaged over 85 genes was not substantially different from the nominal 0.05 level. (Note that the CIs are constructed from the standard deviation of the gene-specific type I error estimates, so tend to be quite narrow). There was slight elevation of empirical type I error for MinP-M, especially for the analysis of only LF SNPs. This likely reflects an inadequacy of the multivariate normal distribution approximation used for correlated multiple testing (Conneely and Boehnke, 2007). The empirical type I error for trait model 1 was slightly inflated across all tests.

Depending on the trait model, the choice of analysis set affected power differently (Figure 2). For Models 1 and 2, analysis using only LF SNPs was most powerful, while analysing only common SNPs was least powerful, and using the combined set yielded power slightly lower than using LF SNPs alone. In contrast, for Model 3, power was somewhat higher using all SNPs, and lowest for the LF SNPs. For Models 4 and 5, which have one common causal SNP and one LF causal SNP, the combined and common SNP sets showed similar power in comparison to lower power in the LF set.

Since the causal SNPs in Models 1 and 2 have low frequency and most genes have at least one LF SNP that is strongly correlated with the causal SNPs, the analysis of LF SNPs alone is usually an efficient choice in terms of *df* and tagging power for causal effect. Although Model 3 also specifies two low frequency causal variants, with the combination of deleterious and protective effects, *a*_1_ = 1 and *a*_2_ = −1, analysis of LF SNPs alone had the lowest power. In this case, a LF SNP that is positively correlated with both causal SNPs will usually appear almost unassociated with the quantitative trait. These observations may be further understood by the expected beta coefficients calculated using equations (1) and (2) (Table 3). The percentage of strongly associated SNPs (|β | > 0.5) is high for LF SNP analysis in Models 1 and 2, but substantially lower for Model 3. Also, the mean of | β | is higher in the LF SNP analysis compared to all SNP or common SNP analysis in Models 1 and 2, whereas it was lower in Model 3.

In Models 4 and 5, however, the two causal SNPs were not required to be within the same bin. So the common causal SNP was more likely to be well-tagged by common SNPs, and analysis of LF SNPs alone had lower power irrespective of whether the LF causal variant was deleterious or protective. The percentages of strongly associated SNPs (|β | > 0.5) in the analysis using all SNPs or common SNPs were both higher for Models 4 and 5 when compared with their counterparts, Models 2 and 3, respectively. However, in the analysis using LF SNPs these percentages were lower for Model 4 compared to Model 2, but higher for Model 5 compared to Model 3, which is consistent with the power results for these models.

### Comparisons among gene-based tests

We compared the performance of gene-based tests for each trait model under the three gene sets analyses. In general, the Wald test was more powerful and robust across different simulation scenarios, while differences in power among the other tests were variable, depending on the scenario (Table 5 and Figures 2–7).

Under Models 1–3 in which the causal SNPs are all LF variants, the Wald test was notably more powerful than other tests when analysing only common SNPs. When we compared the distribution of expected beta coefficients from joint and marginal regression analysis of common SNPs, we found that the percentages of strongly associated SNPs (|β |> 0.5) was high for joint analysis but low for marginal analysis for Models 1–3 (Table 3). Since pairwise correlation is not likely to be strong if the tagging SNP is common and the causal SNP is rare (Figure 8), marginal effects of common SNPs under a LF causal model usually are not strong. Corresponding to these results, the statistics based on marginal analysis of common SNPs such as MinP-M, SSB, SSBw, and SKAT did not perform well for Models 1–3. With the common SNP analysis, the joint regression analysis captured rare causal effects better than marginal analysis, presumably because of the presence of three-way or higher-order LD among the causal SNP and two or more common SNPs.

However, except for the Wald test, the MLC and LC tests based on joint regression did not perform well-under Models 1–3 for common SNPs alone. The sum of β from the joint regression analysis of common SNPs for these models was much smaller than the sum of |β |, suggesting co-occurrence of deleterious and protective associations (Table 3). This gives some insight into the under-performance of MLC and LC tests for common SNP analysis for these models even though the joint regression captures the low frequency causal effect to some degree.

For Model 3 where the causal effects are opposing, the empirical power of MLC tests, MinP-M, PC80, SSB, SSBw, and SKAT with analysis of all SNPs was substantially lower than that of the Wald test, whereas for the other trait models, these tests were more powerful than the Wald when the analysis included all SNPs (Figures 2, 5). The expected beta coefficient for the marginal association was low for both common and rare SNPs, which resulted in relatively low power for the tests based on marginal analysis (Table 3). The joint analysis captured the causal effect better than the marginal analysis for the case of Model 3, but neither of the MLC or LC tests perform well since the captured effects are opposing as indicated by the sum of β near to zero. Model 3 is essentially a “worst case” for the MLC test construction because the opposing LF SNPs are positively correlated and are assigned to the same bin.

For Models 4 and 5 where two causals were in different frequency groups, and therefore usually in different bins, MLC tests performed best for the analysis using both common and LF SNPs (Figures 2, 6, 7). This can most likely be explained by reduced *df* and low prevalence of opposing effects for MLC tests, while the effects of the two causal SNP from both frequency groups are captured well.

## Discussion

In this study, we examined the performance of several multi-marker methods that can be applied to combined analysis of common and low frequency variants. Using 85 different gene panels which include many low frequency SNPs, we simulated trait models with untyped low frequency causal SNPs. Moreover, by calculating the expected beta estimates of indirect association for joint and marginal regression analysis, we provide some insight into the performance of gene-based statistics in different situations.

In our comparison of different analysis sets of SNPs, we found that combined analysis of low frequency and common SNPs together is a robust choice that works for various trait models whereas analysis using only common SNPs or only low frequency SNPs can lose power in certain situations. The good performance of multi-marker tests using a combined set of SNPs is not surprising when one of the causal SNPs is common and the other is rare. Also, when causal SNPs consist of only low frequency variants, it is natural to expect better performance in analysis of only low frequency SNPs due to smaller *df* and correlation between typed/analysed SNPs and untyped causal SNPs, but the reduction in power incurred in the combined set of SNPs was rarely very large. Furthermore, for the trait model in which the causal effects are opposing (one deleterious, and one protective), analysis using the combined set of SNPs was a better choice.

Across the different trait models we investigated, the statistic that showed the most robust performance was the Wald test. In our previous study of the MLC and other gene-based statistics, MLC tests, MinP-M, PC80, and SSB tests using common SNPs usually performed better than the Wald test for trait models based on common causal SNPs (Yoo et al., 2013). In this study, the occasional poor performance of statistics based on marginal tests for low frequency causal variant trait model occurred when the marginal regression analysis failed to capture the low frequency SNP effects due to lower correlation with the causal SNP (see Figure 8). The reason for poor performance of MLC tests differed from that of tests based on marginal regression analysis. Since the joint regression analysis was usually better in capturing low frequency causal effects due to multilocus LD, the Wald test performed well. However, the effects captured by multiple SNPs were mostly in opposing directions and since the SNPs had high positive correlation, and fell within the same bin, the MLC tests usually suffered.

The genotypes in our simulation were derived from HapMap haplotypes, and therefore are expected to represent realistic values occurring in an Asian population, at least for more common SNPs. Therefore, the genes selected to include many low frequency correlated SNPs were only 85 in number when we limited the gene size to be between 8 and 15. We expect genotyping data obtained through sequencing study would have a large number of low frequency correlated SNPs and more diversity in gene structure. Further simulation studies based on sequencing data might be needed to address realistic gene structure in a broad sense. Along the same lines, it would be of interest to evaluate the use of imputed SNPs for multi-marker tests. If we could remove or reduce the bias caused by the omitted causal SNPs and use proper global tests for the imputed SNPs, more powerful analysis may be performed.

Many popular multi-marker tests for rare variants are based on marginal analysis, but we were able to confirm the merit of joint regression analysis for certain trait models. Tests based on joint regression analysis are in need of further development. Joint regression analysis is more suitable for combined analysis of common and low frequency variants in a gene-based analysis framework. Also, further study of a combination of gene-based tests having different merits for different situations would be warranted.

## Author contributions

Yun Joo Yoo participated in design of research, computational analysis, and drafting of the paper. Lei Sun participated in design of research, interpretation of the results, and revising the paper with critical content. Shelley B. Bull participated in design of research, interpretation of the results, and drafting of the paper.

### Conflict of interest statement

The authors declare that the research was conducted in the absence of any commercial or financial relationships that could be construed as a potential conflict of interest.
